# microRNA-501-3p suppresses metastasis and progression of hepatocellular carcinoma through targeting LIN7A

**DOI:** 10.1038/s41419-018-0577-y

**Published:** 2018-05-10

**Authors:** Chubin Luo, Dan Yin, Hao Zhan, Uyunbilig Borjigin, Chuanjiang Li, Zhengjun Zhou, Zhiqiang Hu, Pengcheng Wang, Qiman Sun, Jia Fan, Jian Zhou, Xin Wang, Shaolai Zhou, Xiaowu Huang

**Affiliations:** 1 Department of Liver Surgery and Transplantation, Liver Cancer Institute, Zhongshan Hospital, Fudan University; Key Laboratory of Carcinogenesis and Cancer Invasion of Ministry of Education, 200032 Shanghai, China; 2 Institute of Biomedical Sciences, Fudan University, Shanghai, China; 3 The State Key Laboratory of Reproductive Regulation and Breeding of Grassland Livestock, Inner Mongolia University, 010070 Hohhot, China; 4 Department of Hepatobiliary Surgery, Nanfang Hospital, Southern Medical University, 510515 Guangzhou, China; 5 Hepatoscience, Inc., Sunnyvale, CA 94085 USA; 6 Shanghai Key Laboratory of Organ Transplantation, Shanghai, China

## Abstract

Increasing numbers of evidences have demonstrated that microRNAs (miRNAs) are implicated in metastasis and progression of hepatocellular carcinoma (HCC). However, their detailed expression levels and actual functions in HCCs have not been fully clarified yet. Results from our recent study revealed that some miRNAs were particularly related to metastasis of HCCs. As one of these newly found miRNAs, miR-501-3p showed to highly involve into metastatic process of HCCs. Here we reported that the expression of miR-501-3p was decreased in both metastatic HCC cell lines and tissue samples from HCC patients with recurrence and metastasis. Downregulation of miR-501-3p correlated with tumor progression and poor prognosis in the HCC patients. Results of functional analyses revealed that overexpression of miR-501-3p in HCCLM3 cancer cells inhibited their proliferation, migration, invasion, and epithelial–mesenchymal transition (EMT), while miR-501-3p loss in PLC/PRF/5 cancer cells facilitated all these cellular activities. In addition, Lin-7 homolog A (LIN7A) was directly targeted by miR-501-3p to mediate the suppression effects on metastasis in HCC cells. miR-501-3p suppresses metastasis and progression of HCCs through targeting LIN7A. This finding suggests that miR-501-3p could be used as a potential prognostic predictor as well as a potential therapeutic tool for HCC therapies.

## Introduction

Among the most common malignant cancers, hepatocellular carcinoma (HCC) ranks number five and has become the second cause of cancer death over the world^1^. Although remarkable advances have been reached in surgical techniques and perioperative managements, the prognosis of HCC patients after hepatectomy is still unsatisfactory because of the high rates of intrahepatic and/or distal metastasis as well as progression^2^. Presently, the urgent necessity is to figure out the molecular mechanisms relative to the metastasis and progression in HCCs and to find the biomarkers for prognostic prediction and novel targets for HCC therapies.

MicroRNAs (miRNAs) comprise a class of small noncoding RNAs that are endogenously expressed to regulate gene expression levels through binding to the 3′-untranslated region (UTR) of their target mRNAs for inducing their cleavages or translational repressions subsequently^3, 4^. miRNAs play the critical roles in HCC biological progression by affecting cell proliferation, apoptosis, drug resistance, and metastasis^5–8^. Previous studies confirmed that miR-122, miR-223, miR-124, and miR-203 suppressed tumor growth and metastasis, whereas miR-101, miR-130b, miR-221, miR-21, and miR-222 promoted tumor development in HCCs^9–16^.

In our recent study, miRNA sequencing was performed in several HCC models, including MHCC97L, MHCC97H, and HCCLM3 cancer cell lines as well as the lung metastatic tissues derived from HCCLM3-RFP xenograft model^5^. MHCC97L, MHCC97H, and HCCLM3 cells were all established in our previous studies, which showed the step-wisely increased potential of metastasis with the same genetic background but carried different metastatic potentials after xenograft in the lungs^17^. Based on the information of step-wisely increased potential of metastasis in them and the xenografted metastatic tissues in the lung, several particular miRNAs were selected for their involvements in HCC metastasis. Remarkably, miR-501-3p highly associated with metastatic potential of HCCs for its correlation with the step-wisely increased potential of metastasis. However, both the expression of miR-501-3p in HCCs and its potential roles for tumor metastasis and progression had not been known completely. In the present study, the expression levels of miR-501-3p were rigorously studied in several relative HCC cancer cell lines with different metastatic potentials. The expression levels of miR-501-3p were also detected in HCC tissue samples to particularly evaluate its prognostic significance in HCCs. Next, miR-501-3p was further studied for its roles and potential mechanism in tumor metastasis and progression both in vitro and in vivo.

## Results

### Loss of miR-501-3p coincided with metastasis and prognosis of HCCs

**Fig. 1:**
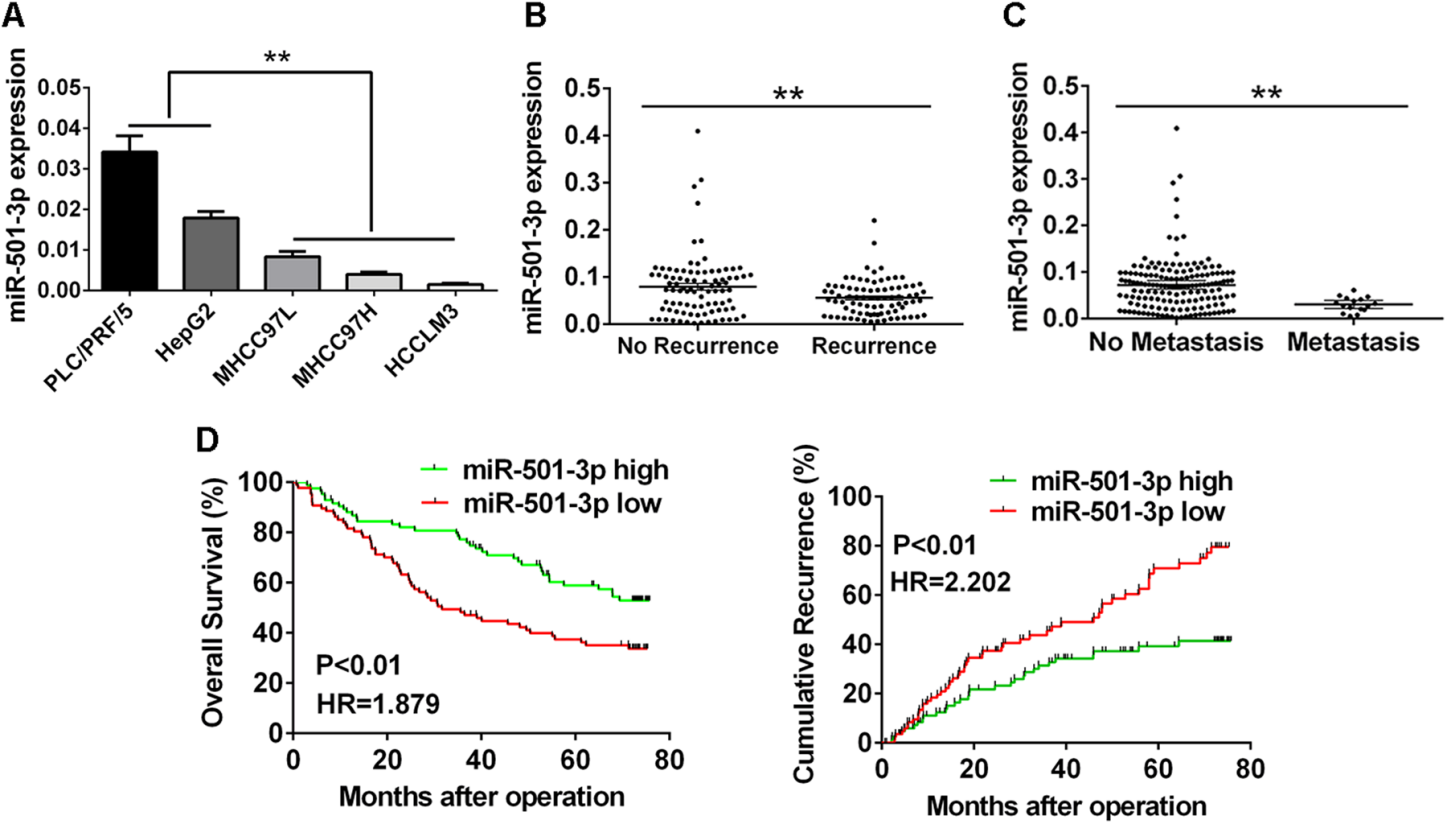
Loss of miR-501-3p coincided with metastasis and prognosis of HCCs. **a** Expression of miR-501-3p in five established HCC cell lines was determined by qRT-PCR. **b** Expression of miR-501-3p in HCC tissues with or without recurrence after surgical resection was determined by qRT-PCR. **P < 0.01. **c** Expression of miR-501-3p in HCC tissues with or without metastasis after surgical resection was determined by qRT-PCR. **P < 0.01. **d** Kaplan–Meier’s analysis showed that HCC patients with overexpression of miR-501-3p exhibited better overall survival and a lower cumulative recurrence rate compared with those who had low expression of miR-501-3p. Data depicts the mean ± standard deviation and are representative of three independent experiments. HR hazard ratio

At first, the expression level of miR-501-3p was studied for its correlation with different metastatic potentials in several HCC cell lines. Results revealed that the expression level of miR-501-3p decreased in all the analyzed metastatic HCC cell lines (MHCC97L, MHCC97H, and HCCLM3), in comparison with the non-metastatic HCC cell lines (PLC/PRF/5 and HepG2) (Fig. 1a). Next, the expression level of miR-501-3p was also detected in HCC specimens (*n* = 171). Results indicated that miR-501-3p expression was significantly lower in HCC patients with recurrence than those patients without recurrence (Fig. 1b). Expression level of miR-501-3p also decreased in HCC patients with metastasis, when compared to those without metastasis (Fig. 1c). In addition, the relationship between miR-501-3p level and several clinicopathological features was examined in HCC patients. Results indicated that the decrease of miR-501-3p expression level was significantly correlated with the parameters of tumor size (*P* = 0.040), microvascular invasion (*P* = 0.033), and TNM stage (*P* = 0.011) (Table 1). However, miR-501-3p expression level was not significantly correlated with other clinical characteristics, including age, sex, hepatitis B surface antigen, preoperative alpha-fetoprotein, gamma glutamyl transferase, cirrhosis, tumor number, tumor encapsulation, or tumor differentiation (Table 1). Results of Kaplan–Meier analysis showed that the 1-, 3-, and 5-year overall survival (OS) rates for HCC patients with low miR-501-3p expression levels (81.6, 48.3, and 37.5%, respectively) significantly reduced, when compared with the patients with high miR-501-3p expression levels (88.1, 77.2, and 58.9%, respectively). Moreover, the 1-, 3-, and 5-year cumulative recurrence rates in HCC patients with low miR-501-3p expression levels (18.4, 45.5, and 70.8%, respectively) significantly increased, when compared with HCC patients with high miR-501-3p expression levels (12.4, 31.4, and 39.3%, respectively) (Fig. 1d). Thus our findings indicated that downregulation of miR-501-3p might play a potential role to promote the malignant progression of HCCs.

**Table 1:** Correlation between the clinicopathologic characteristics and miR-501-3p expression in HCCs (*n*=171)

Clinical parameters	Cases	miR-501-3p levels		*P*-value
		Low	High	
Age (years)				
≤50	82	47	35	0.106
>50	89	40	49	
Gender				
Male	148	78	70	0.226
Female	23	9	14	
HBsAg				
Positive	152	80	72	0.194
Negative	19	7	12	
AFP (ng/ml)				
≤400	115	59	56	0.873
>400	56	28	28	
GGT (U/L)				
≤54	71	34	37	0.510
>54	100	53	47	
Cirrhosis				
No	26	15	11	0.450
Yes	145	72	73	
Tumor size (cm)				
≤5	78	33	45	0.040*
>5	93	54	39	
Tumor number				
Solitary	143	70	73	0.255
Multiple	28	17	11	
Microvascular invasion				
Absent	100	44	56	0.033*
Present	71	43	28	
Tumor encapsulation				
Absent	61	27	34	0.198
Present	110	60	50	
Tumor differentiation				
I/II	137	68	69	0.514
III/IV	34	19	15	
TNM stage				
I/II	130	59	71	0.011*
III/IV	41	28	13	

Chi-square test was used in all analysis

*HBsAg* hepatitis B surface antigen, *AFP* alpha-fetoprotein, *GGT* gamma glutamyl transferase, *TNM* tumor–node–metastasis

*P<0.05

Bold values signify *P*-value <0.05

### miR-501-3p inhibited proliferation, migration, and invasion of HCC cells in vitro

**Fig. 2:**
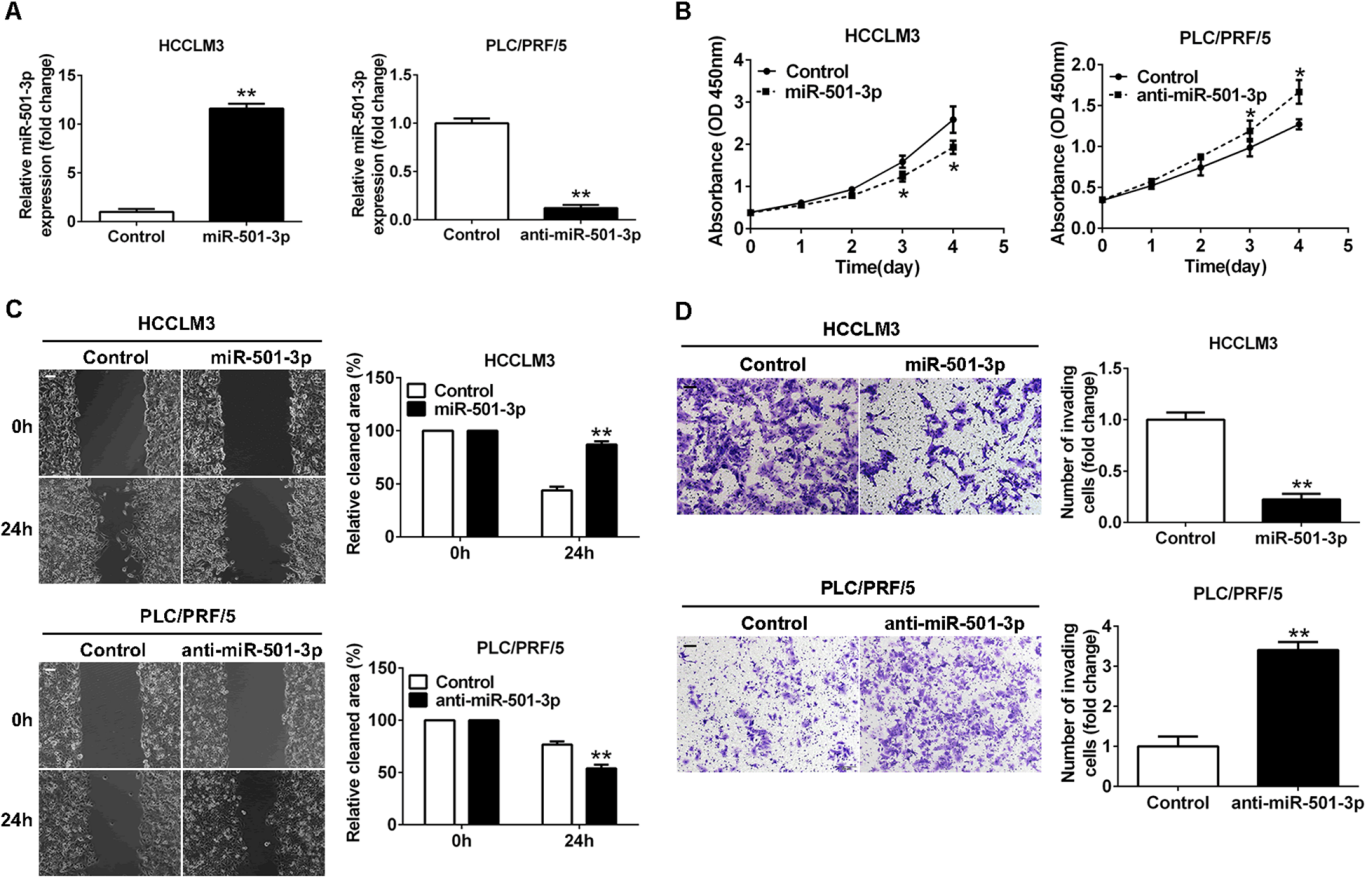
miR-501-3p inhibits the proliferation, migration and invasion of HCC cells in vitro. **a** Expression of miR-501-3p in HCCLM3 and PLC/PRF/5 cells that were transfected with corresponding vectors was determined by qRT-PCR. **b** CCK-8 assay was performed to detect cell proliferation. *P < 0.05. **c** Wound-healing migration assays and the quantification of the percentage of open area were shown. Scale bar: 100 μm, **P < 0.01. **d** Invasive behavior was tested using transwell invasion assays after overexpression or knockdown of miR-501-3p in HCC cells. Scale bar: 100 μm, **P < 0.01. Data depicts the mean ± standard deviation and are representative of three independent experiments

To explore the functional role of miR-501-3p in HCCs, both gain and loss-of-function experiments were performed in HCCLM3 and PLC/PRF/5 cell lines, which had the different levels of miR-501-3p. Known from quantitative reverse transcriptase–polymerase chain reaction (qRT-PCR) assay, the expression of miR-501-3p in HCCLM3 cells was successfully overexpressed by the stable infection of miR-501-3p lentiviral vectors, while the expression of miR-501-3p in PLC/PRF/5 cells was downregulated by stable infection of anti-miR-501-3p lentiviral vectors (Fig. 2a). Cell Counting Kit-8 (CCK-8) assay indicated that upregulation of miR-501-3p in HCCLM3 cells inhibited cell proliferation, whereas knockdown of miR-501-3p in PLC/PRF/5 cells increased cell proliferation (Fig. 2b). Results of both wound-healing assay and transwell assay revealed that the HCCLM3-miR-501-3p cells had a slower wound-closure rate and less capacity of cell invasions than HCCLM3 cells as control, whereas the PLC/PRF/5-anti-miR-501-3p cells had a higher migratory and more invasive capacity than PLC/PRF/5 cells as control (Fig. 2c, d). Therefore, miR-501-3p played a suppressive role in regulation of proliferation, migration, and invasion in HCC cells.

### miR-501-3p inhibited epithelial–mesenchymal transition (EMT) of HCC cells

**Fig. 3:**
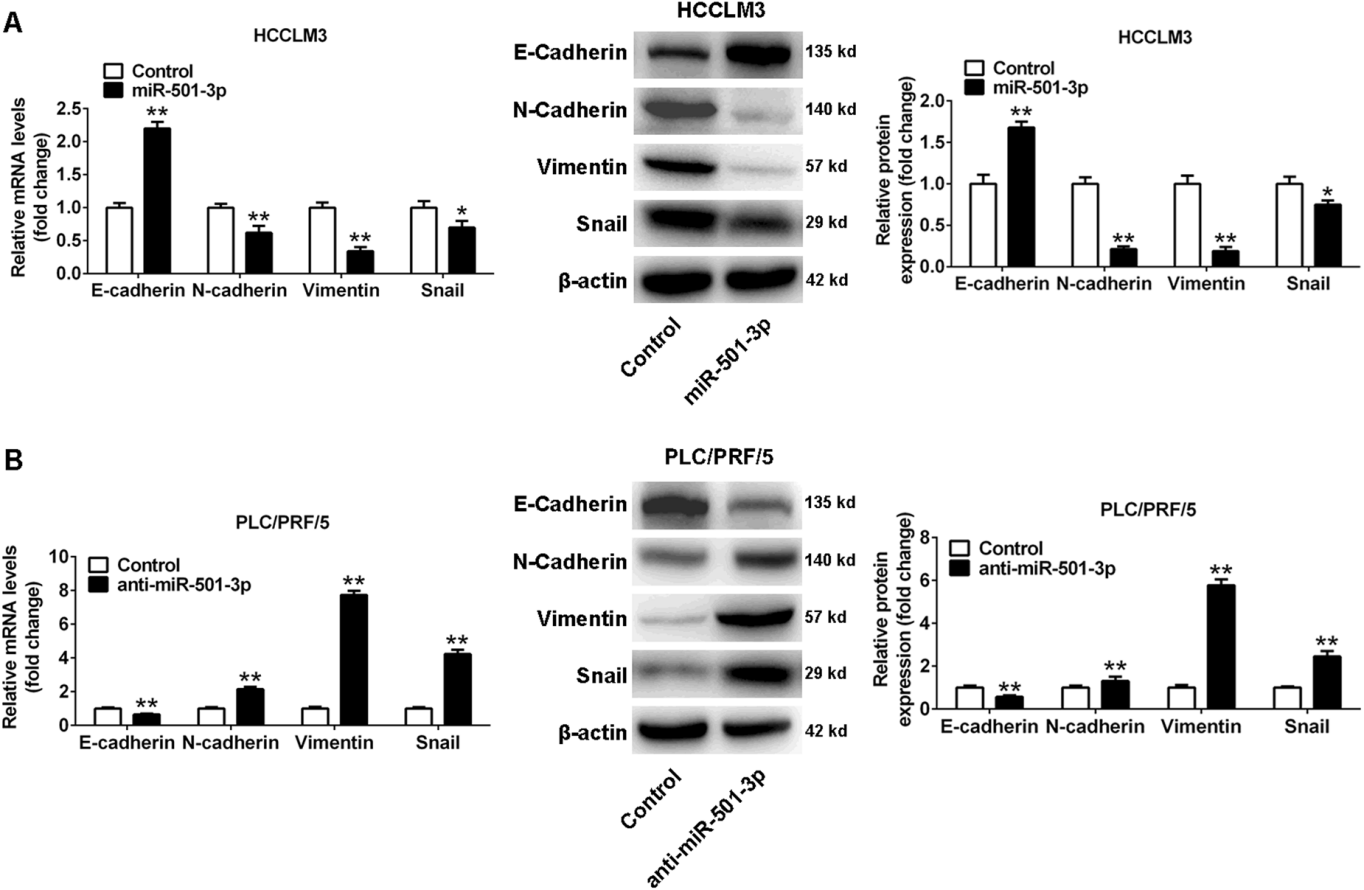
miR-501-3p inhibits EMT in HCC cells. qRT-PCR (**a**) and western blot analysis (**b**) show changes in EMT marker expression following stable upregulation or downregulation of miR-501-3p expression. *P < 0.05, **P < 0.01. Data depicts the mean ± standard deviation and are representative of three independent experiments

Increasing numbers of evidences have suggested that EMT plays a critical role in the metastasis of HCCs. For this, miR-501-3p was studied for inhibition on HCC metastasis via regulating EMT. The expression of EMT markers was detected and results indicated that the level of E-cadherin, as an epithelial marker, was increased after miR-501-3p upregulation in HCCLM3 cells, whereas the levels of mesenchymal markers including N-cadherin, Vimentin, and Snail were all decreased (Fig. 3a). Conversely, inhibition of miR-501-3p in PLC/PRF/5 cells led to a downregulation of E-cadherin but upregulation of N-cadherin, Vimentin, and Snail (Fig. 3b). Therefore, miR-501-3p acted as a suppressor of EMT in HCC cells.

### miR-501-3p suppressed the growth and metastasis of HCCs in vivo

**Fig. 4:**
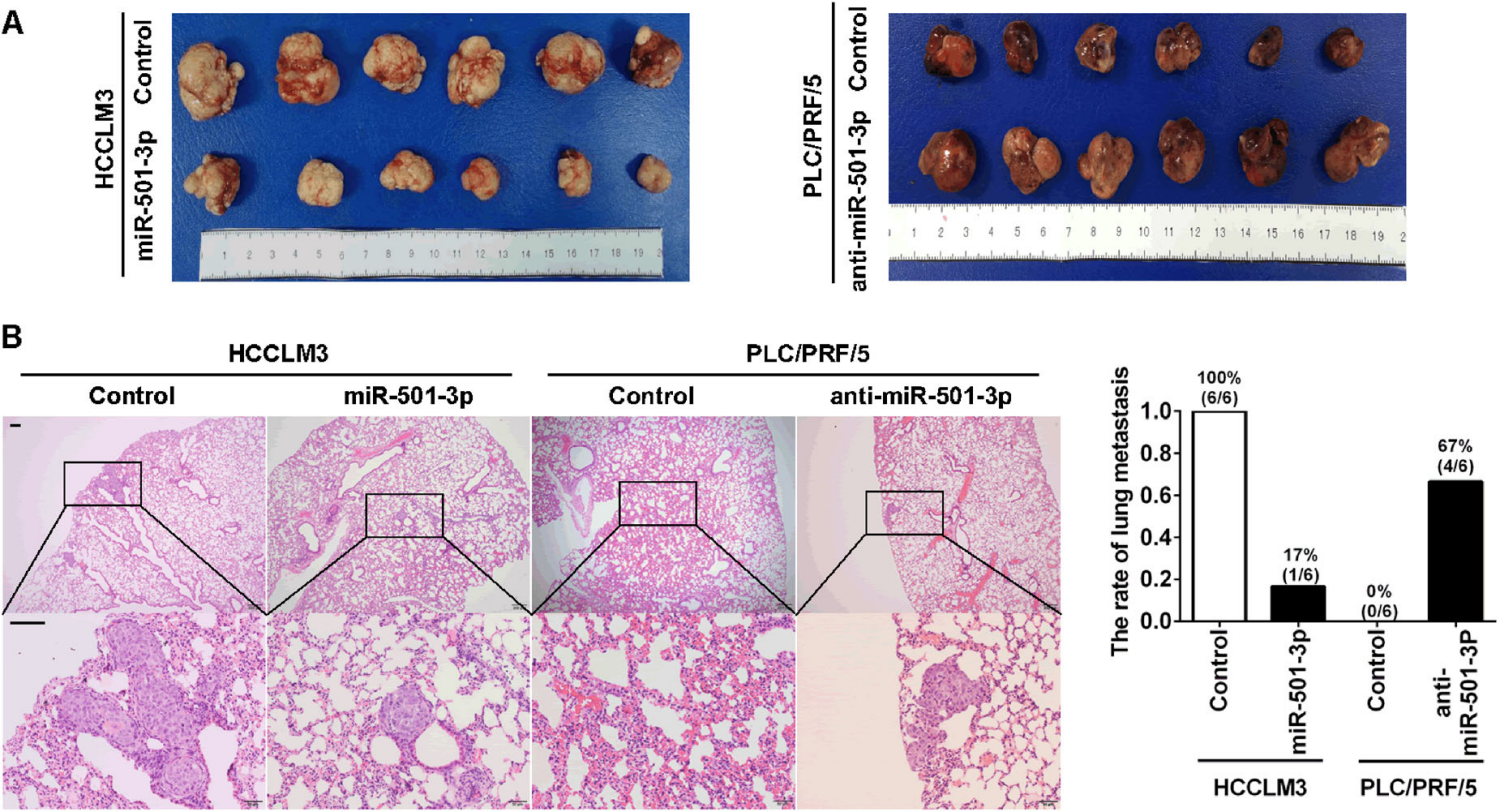
miR-501-3p inhibits HCC growth and metastasis in a xenograft nude mice model. **a** Macrograph of tumors in all groups. **b** Hematoxylin and eosin (H&E)-stained images of lung metastatic nodules from all groups with magnification of the selected areas. Scale bar: 100 μm. Data depicts the mean ± standard deviation and are representative of three independent experiments

Orthotopic HCC mouse models were used to analyze the function of miR-501-3p in vivo. Overexpression of miR-501-3p in HCCLM3 cells (1.84 ± 0.41 cm^3^) resulted in a significant decrease in tumor volume, in comparison with controls (5.16 ± 0.46 cm^3^), while miR-501-3p knockdown in PLC/PRF/5 cells (3.50 ± 0.23 cm^3^) led to a significant increase in tumor volume compared to controls (1.26 ± 0.10 cm^3^) (Fig. 4a). Incidence of pulmonary metastasis was 17% (1 of 6) in HCCLM3-miR-501-3p cell transplanted mice, which was significantly lower than that in HCCLM3 cell transplanted mice (6 of 6). Incidence of pulmonary metastasis of PLC/PRF/5-anti-miR-501-3p cell transplanted mice was 67% (4 of 6), while no pulmonary metastasis occurred in PLC/PRF/5 cell transplanted mice (0 of 6) (Fig. 4b). Thus these data indicated that miR-501-3p inhibited growth and metastasis of HCCs in vivo.

### Lin-7 homolog A (LIN7A) was found as one of the targets for miR-501-3p

**Fig. 5:**
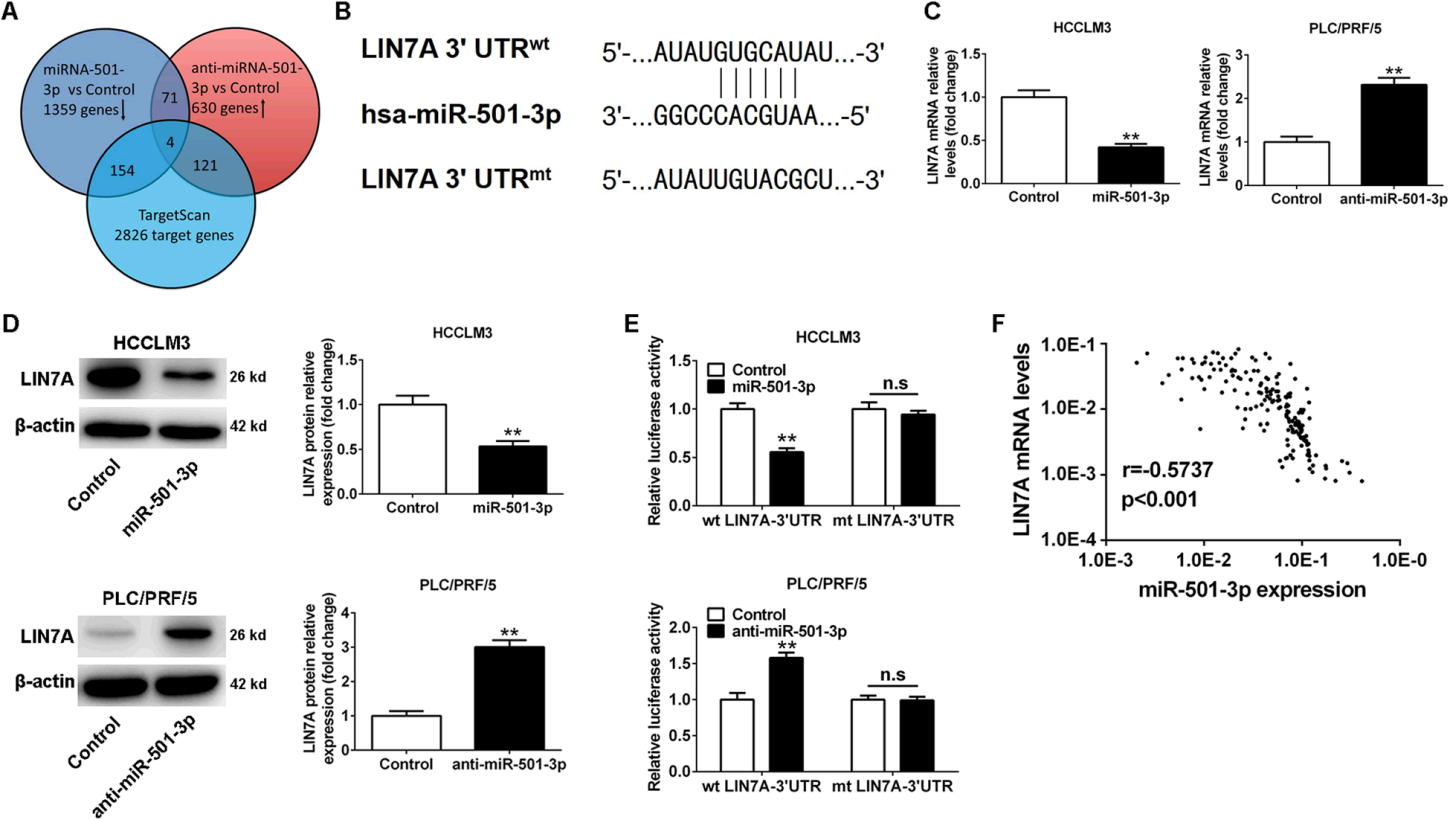
LIN7A is a direct target of miR-501-3p in HCC cells. **a** Venn diagrams showing the number of genes identified as potential targets of miR-501-3p. **b** Diagrams show the miR-501-3p putative binding sites and corresponding mutant sites of LIN7A. **c** HCCLM3 and PLC/PRF/5 cells that were transfected with corresponding miRNA vectors were subjected to qRT-PCR for LIN7A expression. **P < 0.01. **d** miR-501-3p overexpression reduced the expression of LIN7A protein in HCCLM3 cells and miR-501-3p knockdown increased the level of LIN7A protein in PLC/PRF/5 cells. **P < 0.01. **e** miR-501-3p overexpression significantly suppressed, whereas miR-501-3p loss increased the luciferase activity of LIN7A containing a wild-type (wt) of 3′-UTR but not a mutant (mt) 3′-UTR. **P < 0.01; n.s, no significance. **f** An inverse correlation between the levels of miR-501-3p and LIN7A mRNA was observed in HCC tissues. Data depicts the mean ± standard deviation and are representative of three independent experiments

To further investigate the underlying mechanisms for how miR-501-3p exert its functional effects on HCC cells, identification of the target genes for miR-501-3p were performed through manner of overlapped genes in the following groups: (1) downregulated genes in HCCLM3-miR-501-3p cells compared with HCCLM3 cells; (2) upregulated genes in PLC/PRF/5-anti-miR-501-3p cells compared with PLC/PRF/5 cells; and (3) genes predicted as potential targets for miR-501-3p by target prediction algorithm TargetScan (http://www.targetscan.org) (Fig. 5a). CDH6, LIN7A, UGT2B10, and ZFPM1 were found for common involvements among these three groups (Table 2). Among the identified potential targets, LIN7A was first analyzed for its special characters. LIN7A was previously known as a crumbs-complex polarity gene that could regulate tumor progression. Different from other three potential targets, the role of LIN7A in HCCs remained unknown completely. Hence, our subsequent analyses were focused on LIN7A in HCCs. The complementary sequence of miR-501-3p was found in the 3′-UTR of LIN7A mRNA (Fig. 5b). The relationship between miR-501-3p and LIN7A was analyzed in qRT-PCR and western blot assays. Results indicated that overexpression of miR-501-3p significantly decreased levels of both mRNA and protein for LIN7A in HCCLM3 cells, whereas miR-501-3p knockdown increased the levels of both mRNA and protein for LIN7A in PLC/PRF/5 cells (Fig. 5c, d). To further confirm LIN7A as a direct target of miR-501-3p, results of the luciferase reporter gene assay demonstrated that overexpression of miR-501-3p was able to decrease luciferase activity of wild-type (wt) construct of LIN7A 3′-UTR. In contrast, miR-501-3p silencing increased luciferase activity of wt construct of LIN7A 3′-UTR. However, no significant change of the luciferase activity was found with mutant (mt) construct of LIN7A 3′-UTR after modulating the miR-501-3p expression (Fig. 5e). Significantly, the mRNA levels of LIN7A negatively correlated with the miR-501-3p expression in HCC tissues (Fig. 5f). Taken together, our data suggested that LIN7A could be a direct downstream target of miR-501-3p in HCCs.

**Table 2:** Changed expression levels of the predicted genes in HCC cells upon miR-501-3p or anti-miR-501-3p treatment

miR-501-3p vs Control	Fold change (low to high)	Anti-miR-501-3p vs Control	Fold change (high to low)
LIN7A	0.34	LIN7A	2.95
CDH6	0.42	CDH6	2.37
UGT2B10	0.38	UGT2B10	2.08
ZFPM1	0.43	ZFPM1	2.72

### LIN7A mediated the effects of miR-501-3p on HCC cells

**Fig. 6:**
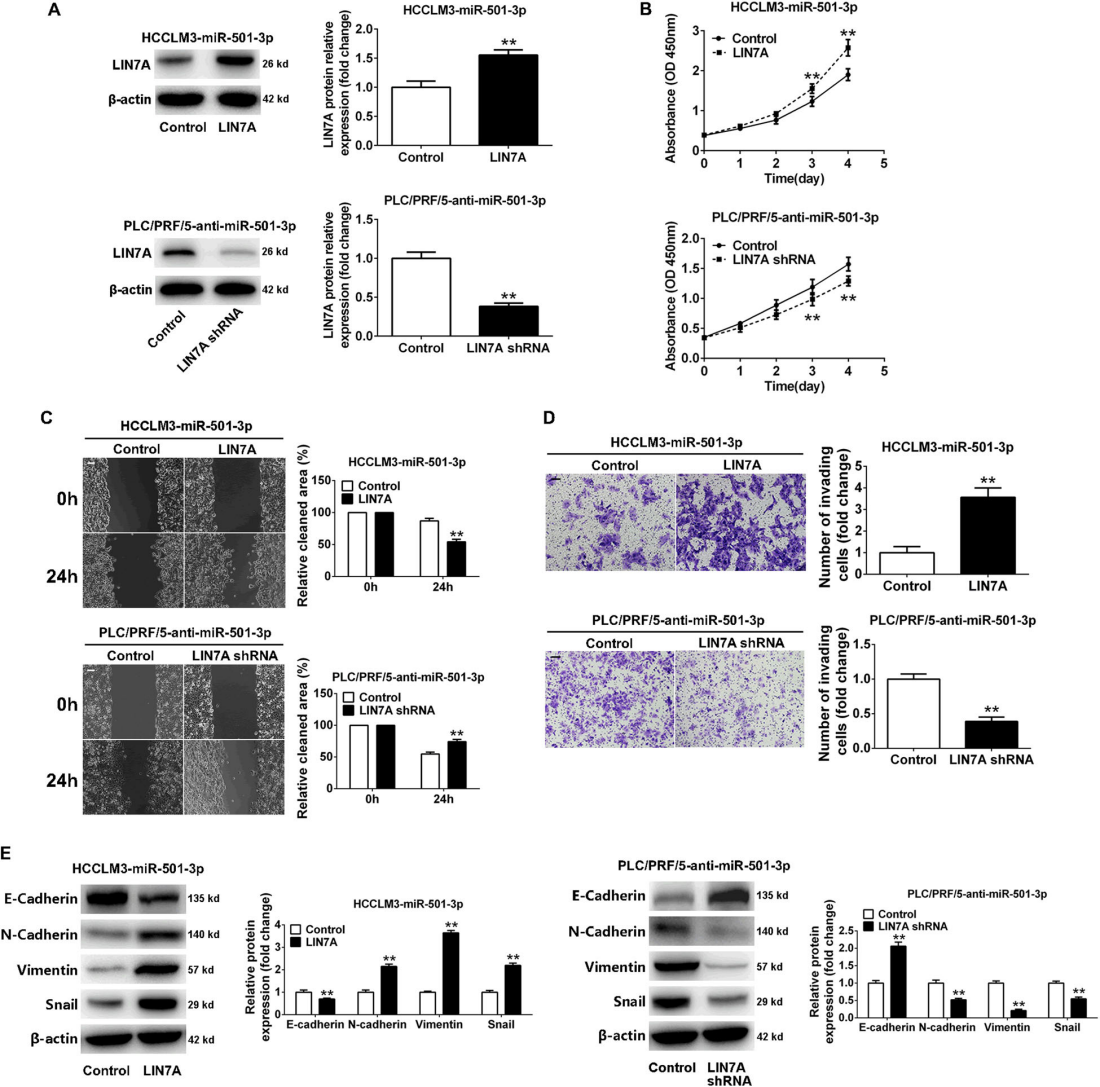
Modulation of LIN7A expression abolished miR-501-3p-mediated cellular activities in vitro. **a** Western blot showed the expression of LIN7A protein in HCCLM3-miR-501-3p cells that were transfected with LIN7A vector or PLC/PRF/5-anti-miR-501-3p cells that were transfected with shLIN7A vector and their corresponding controls. **P < 0.01. **b** Proliferation of HCCLM3-miR-501-3p that were transfected with LIN7A and PLC/PRF/5-anti-miR-501-3p cells that were transfected with shLIN7A vector and their corresponding controls was determined by CCK-8 assay. **P < 0.01. **c** Wound-healing assay and **d** transwell assay were performed to determine the effects of LIN7A on the migration and invasion of HCC cells. Scale bar: 100 μm, **P < 0.01. **e** LIN7A restoration decreased the expression of E-cadherin and increased the levels of N-cadherin, Vimentin, and Snail in miR-501-3p-overexpressing HCCLM3 cells. LIN7A knockdown abolished the effects of miR-501-3p loss on EMT process of PLC/PRF/5 cells. **P < 0.01. Data depicts the mean ± standard deviation and are representative of three independent experiments

Next, rescue experiments were performed to confirm whether miR-501-3p executed its functional effects by suppressing its target genes. LIN7A expression was restored after the overexpression of miR-501-3p in HCCLM3-miR-501-3p cells (Fig. 6a). Restoration of LIN7A expression blocked the miR-501-3p-mediated inhibitory effects on proliferation, migration, and invasion in HCCLM3 cells. On the other hand, silencing of LIN7A expression by a specific short hairpin RNA (shRNA) resulted in a reverse effect, which led to reverse the promoting function of miR-501-3p loss on PLC/PRF/5 cells (Fig. 6b–d). However, modulating CDH6, UGT2B10, and ZFPM1 expression levels did not affect the miR-501-3p-mediated effects in HCC cells (data not show). In addition, the ectopic expression of LIN7A attenuated the effect of EMT inhibition in HCCLM3-miR-501-3p cells, whereas knockdown of LIN7A resulted in a reverse effect on EMT inhibition in PLC/PRF/5-anti-miR-501-3p cells (Fig. 6e). Therefore, these data demonstrated that LIN7A acted as a functional downstream target of miR-501-3p in HCC cells.

### LIN7A mediated the effects of miR-501-3p in vivo

**Fig. 7:**
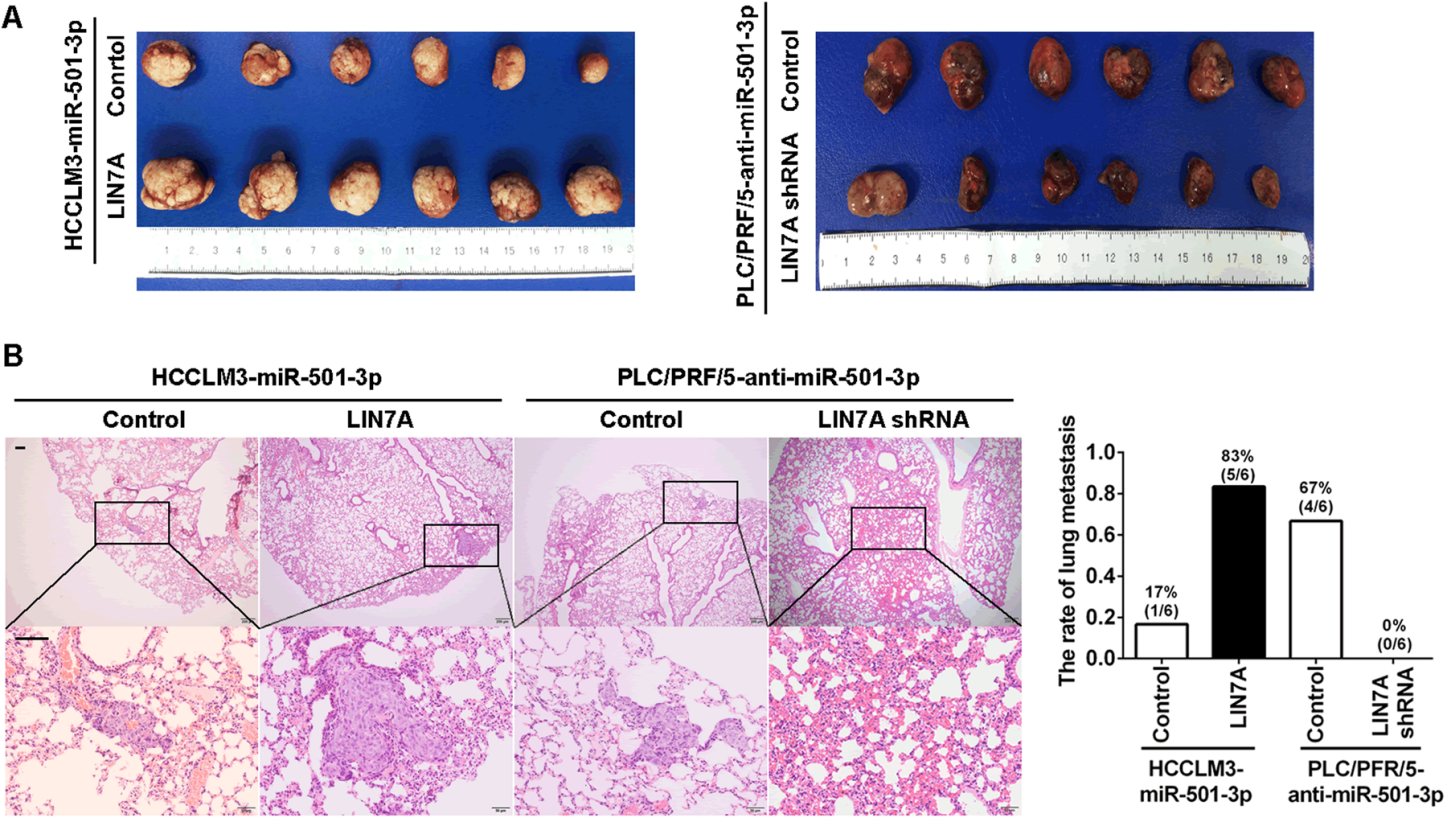
Modulation of LIN7A expression abolished miR-501-3p-mediated functions in vivo. **a** Macrograph of tumors in all groups. **b** H&E-stained images of metastatic nodules in the lungs from all groups with magnification of the selected areas. Scale bar: 100 μm. Data depicts the mean ± standard deviation and are representative of three independent experiments

The roles of miR-501-3p-LIN7A signaling on modulating tumor growth and metastasis were examined in vivo. Restoration of LIN7A expression in HCCLM3-miR-501-3p cells (4.17 ± 0.48 cm^3^) resulted in a significant increase in tumor volume, in comparison with controls (1.76 ± 0.27 cm^3^), counteracting the suppression of tumor volume induced by overexpression of miR-501-3p in HCCLM3 cells. Knockdown of LIN7A in PLC/PRF/5-anti-miR-501-3p cells (1.17 ± 0.20 cm^3^) led to a significant decrease in tumor volume compared to controls (3.08 ± 0.34 cm^3^), reversing the promoting effect induced by miR-501-3p loss in PLC/PRF/5 cells (Fig. 7a). Pulmonary metastasis occurred in 83% (5 of 6) of HCCLM3-miR-501-3p-LIN7A cell transplanted mice, which was significantly higher than that in HCCLM3-miR-501-3p cell transplanted mice (1 of 6). No pulmonary metastasis occurred in PLC/PRF/5-anti-miR-501-3p-shLIN7A cell transplanted mice (0 of 6), while incidence of pulmonary metastasis of PLC/PRF/5-anti-miR-501-3p cell transplanted mice was 67% (4 of 6) (Fig. 7b). These results suggested that LIN7A mediated the inhibitory effects of miR-501-3p in HCCs in vivo.

## Discussion

Metastasis and recurrence account for the most common mortality in HCC patients after surgical resections. Presently, the urgent necessities are to understand the mechanisms underlying HCC metastasis and to target the metastatic mechanisms for providing more effective treatments of HCCs. Although several studies have demonstrated the roles of miRNAs in metastasis and progression of HCCs^18–21^, the underlying molecular mechanisms by which miRNAs regulate HCC metastasis and progression are still not clarified. Hence, it is critical to uncover the regulatory characteristics of miRNAs in regulation of HCC metastasis and progression for elucidating miRNAs as potential biomarkers for clinical diagnosis and prognosis and developing novel potential therapeutic strategies.

In our recent study, we have finished the initial study to explore the unknown metastasis-related miRNAs in HCCs^5^. We discovered that miR-501-3p was highly correlated with metastatic potential of HCCs. Here we focused on study of this newly identified miRNA in order to explore its roles in HCC metastasis. Our data demonstrated that the expression level of miR-501-3p was lower in metastatic HCC cell lines than in non-metastatic cell lines. Clinically, the levels of miR-501-3p in tumor tissues of HCC patients with metastasis or recurrence were lower than those of patients without. Downregulation of miR-501-3p significantly correlated with adverse clinical features of HCC patients. In addition, survival analyses were performed to reveal that low expression level of miR-501-3p predicted a reduced OS and high cumulative recurrence in HCC patients. These clinical evidences suggested that miR-501-3p could be involved in HCC metastasis and progression and might become a promising prognostic indicator in HCCs. Differently, The Cancer Genome Atlas (TCGA) data suggest that: (1) there is no difference in the miR-501 expression in terms of tumor–node–metastasis (TNM) stage HCCs; and (2) Pearson correlation coefficient between miR-501 and LIN7A is not significant enough. Although possible discrepancy exists between data of TCGA and our present findings, the differences might stem from the different etiologies and racial differences between data from TCGA and our studies. Therefore, the issues relative to etiologies and racial differences will be further analyzed for HCCs in our future studies for miR-501-3p and LIN7A.

Following the identification of prognostic significance of miR-501-3p in HCC clinical samples, its biological roles were analyzed in HCC cell lines. The functional studies were performed to reveal whether miR-501-3p mediated HCC cells' growth and metastasis. Results indicated that upregulation of miR-501-3p inhibited HCC progression by suppression of proliferation and invasion in vitro, whereas miR-501-3p knockdown facilitated these activities. The effect of miR-501-3p in vivo was also, as expected, in agreement with the biological effects of miR-501-3p in vitro. Overexpression of miR-501-3p resulted in a significant decrease in tumor volume and developed less lung metastasis in mice, whereas miR-501-3p knockdown yielded the opposite effects. Tremendous studies have previously revealed that EMT was a key process in promoting HCC invasion and metastasis, during which dysregulation of miRNAs are regarded to play an important role in EMT modulation^22–25^. Therefore, whether and how miR-501-3p mediates HCCs via regulation of EMT process was specially analyzed. Our data showed that overexpression of miR-501-3p resulted in inhibition of EMT in HCC cells, whereas knockdown of miR-501-3p showed an opposite effect. These data suggested that miR-501-3p acts as a metastatic suppressor in HCC. The exact mechanism that governs miR-501-3p expression in HCCs remains to be further investigated for this may help to shed light on the treatments to restore its expression in tumor cells.

Next, the underlying molecular mechanisms were to realize how miR-501-3p mediate metastasis and progression of HCCs. Especially, the potential downstream targets of miR-501-3p that are located in HCCs. By gene expression profiles and bioinformatics analysis, several potential targets of miR-501-3p were identified, including CDH6, LIN7A, UGT2B10, and ZFPM. As a crumbs-complex polarity gene, LIN7A was particularly analyzed given that polarity deficiency is recognized as an essential step of the EMT progression and as a hallmark of tumor invasion and metastasis^26–28^. A recent study also demonstrated that overexpression of LIN7A facilitated proliferation, invasion, and absence of lumen formation in breast tumor cells^29^. However, the role of LIN7A in HCCs has not been known yet. Here our study confirmed that only LIN7A was a direct functional target of miR-501-3p in HCC cells. At first, our data indicated that miR-501-3p could inhibit the levels of both mRNA and protein of LIN7A in HCC cells. Next, results of the luciferase reporter gene assay demonstrated that ectopic expression of miR-501-3p significantly reduced the activity of a luciferase reporter containing the 3′-UTR sequence of LIN7A, while miR-501-3p silencing increased the luciferase activity. Furthermore, the expression levels of miR-501-3p and LIN7A were inversely correlated in HCC tissues. To verify whether LIN7A mediated the effects of miR-501-3p in HCCs, rescue experiments were performed to examine the roles of LIN7A in cellular proliferation, invasion, and EMT. Our data indicated that restoration of LIN7A expression antagonized the effects of miR-501-3p in HCCs both in vitro and in vivo, whereas knockdown of LIN7A mimicked the effects of miR-501-3p overexpression, suggesting that LIN7A is a predominant functional target of miR-501-3p in these processes. In future, it will be interesting to identify the key downstream signaling pathways of LIN7A in HCC cells and their relevance to cancer metastasis and progression.

Taken together, to our knowledge, we presented the first evidence that miR-501-3p expression was reduced in metastatic HCC cell lines and its downregulation related to poor prognosis of HCC patients as well as the clue that miR-501-3p inhibited proliferation and EMT-mediated invasion and metastasis of HCC cells by suppression of LIN7A. Understanding the roles of miR-501-3p involved in HCC metastasis and progression will enable us to use it as a potential prognostic indicator and a therapeutic tool in treating HCCs.

## Materials and methods

### Information of patients and their fellow-up

From January 2010 to May 2010, a total of 171 HCC tissues from patients with HCC undergoing surgical resection were gathered from our institute. All tissue samples were collected immediately after surgical resection and stored at −80°C. Patients involved in this study did not receive any antitumor therapies before surgery. Patients were monitored until April 14, 2016 after surgery. Pathological diagnosis was based on the World Health Organization criteria. Assessment of tumor differentiation grade was based on the classification proposed by Edmondson and Steiner^30^. Assessment of liver function was in accordance with the Child–Pugh scoring system. Tumor stage was determined in conformity to the TNM classification system proposed by the 2010 International Union Against Cancer. Human materials involved in this study were obtained with informed consent from each patient and approved by the ethics committee of Zhongshan Hospital of Fudan University (Shanghai, China). Surveillance of postsurgical patient was described in a previous study^31^. OS was defined as the time between surgery and death or between surgery and the last observation time. The OS data were censored at the last follow-up for surviving HCC patients. Time to recurrence^32^ was defined as the time between the surgical resection and the date of any relapse, both intrahepatic recurrence and extrahepatic metastasis included.

### Cell lines and animals

MHCC97L, MHCC97H, and HCCLM3 cell lines were previously established in our laboratory. HepG2 and PLC/PRF/5 cell lines were obtained from the cell bank of Chinese Academy of Sciences (Shanghai, China). These cells were stored in liquid nitrogen and maintained in Dulbecco’s modified Eagle’s medium (Invitrogen, Carlsbad, USA) containing 10% fetal bovine serum (Invitrogen) in 5% CO_2_ at 37 °C.

Four-week-old male BALB/c nude mice were obtained from Shanghai Institute of Material Medicine and were all raised in specific pathogen-free conditions. Animal care was in accordance with the criteria in the “Guide for the Care and Use of Laboratory Animals” published by the National Institutes of Health (NIH publication 86–23 revised 1985).

### Vectors and cell transfections

miR-501-3p ectopic expression level lentiviral vector (hU6-MCS-Ubiquitin-EGFP-IRES-puromycin-miR-501-3p) and miR-501-3p knockdown lentiviral vector (hU6-MCS-Ubiquitin-EGFP-IRES-puromycin-anti-miR-501-3p) as well as their negative control were purchased from GeneChem (Shanghai, China). LIN7A expression vector (CMV-MCS-SV40-Neomycin-LIN7A) and shRNA LIN7A vector (hU6-MCS-CMV-SV40-Neomycin-shLIN7A) as well as their negative control were also purchased from GeneChem (Shanghai, China). G418 (Sigma-Aldrich, USA) and puromycin (Sigma-Aldrich, USA) was used to select stably transfected clones in accordance with the manufacturer’s protocol, and validation was performed by qRT-PCR and western blot assays. wt or mt 3′-UTR sequence of LIN7A were inserted into pGL3-promoter vector (Promega, Madison, WI). All transfections were performed as previously described^33^.

### RNA isolation and qRT-PCR

RNA isolation and qRT-PCR were performed as described previously^5^. Trizol reagent (Invitrogen, CA, USA) was used to extract total RNA from cell lines and frozen tumor specimens. Synthesis of complementary DNA (cDNA) was performed by TaqMan MicroRNA Reverse Transcription Kit (Applied Biosystems, Foster City, CA) or PrimeScript reverse transcriptase reagent kit (Takara, Osaka, Japan). U6 or glyceraldehyde 3-phosphate dehydrogenase (GAPDH) was used as an internal control. The expression levels of miR-501-3p and LIN7A were, respectively, normalized by U6 and GAPDH to produce a 2^−DDCt^ value for relative expression. HCC patients with miR-501-3p level lower or higher than the mean value of the miR-501-3p level were defined as miR-501-3p^low^ or miR-501-3p^high^, respectively. Taqman probes (2435 for miR-501-5p, 1973 for U6 (Applied Biosystems)) were used for qRT-PCR detection. The other primers used are as follows: LIN7A sequence of forward primer and reverse primer: 5′-GCAACAGCAAAGGCAACAGT-3′ and 5′-CTCTTTTGAGGCCTCCGTGT-3′; E-cadherin sequence of forward primer and reverse primer: 5′-CGAGAGCTACACGTTCACGG-3′ and 5′-GGGTGTCGAGGGAAAAATAGG-3′; N-cadherin sequence of forward primer and reverse primer: 5′-TGCGGTACAGTGTAACTGGG-3′ and 5′-GAAACCGGGCTATCTGCTCG-3′; vimentin sequence of forward primer and reverse primer: 5′-TGCCGTTGAAGCTGCTAACTA-3′ and 5′-CCAGAGGGAGTGAATCCAGATTA-3′; Snail sequence of forward primer and reverse primer: 5′-TCGGAAGCCTAACTACAGCGA-3′ and 5′-AGATGAGCATTGGCAGCGAG-3′; and GAPDH sequence of forward primer and reverse primer: 5′-CTGGGCTACACTGAGCACC-3′ and 5′- AAGTGGTCGTTGAGGGCAATG-3′.

### Western blot assay

The detailed procedures for western blot were previously described^34^. In brief, total proteins were subjected to 10% sodium dodecyl sulfate-polyacrylamide gel electrophoresis and then transferred to polyvinylidene difluoride membranes. The membranes were incubated with primary antibodies and horseradish-peroxidase–conjugated secondary antibodies. Enhanced chemiluminescence assays were used to detect the signal. LIN7A primary antibody was obtained from Abcam (ab174297, Cambridge, MA, USA). E-cadherin, N-cadherin, Vimentin and Snail primary antibodies were purchased from Cell Signaling Technology (Beverly, MA, USA). β-Actin (2103, Sigma-Aldrich, USA) was used as the protein-loading control.

### Cell proliferation, wound-healing and transwell invasion assay

The proliferation of HCC cells was measured using CCK-8 assay. Cells (2 × 10^3^/well) were dispensed into a 96-well plate with 100 μl fresh medium. A total of 10 μl CCK-8 solution (Dojindo, Tokyo, Japan) was put into the cell and incubated for further 2 h. The absorbance was measured at 450 nm. Wound-healing assay was performed to evaluate cell migration. Briefly, appropriate cells were seeded into 35-mm dishes and cultured to form a tight monolayer. A scraped line was created with a 10-μl pipette tip and the remaining cells were incubated for 24 h with no serum-containing culture medium. Photographs of cellular migration toward the scratched area were taken after 24 h and the percentage of open area was assessed. For the transwell invasion assays, matrigel-precoated 24-well and 8-μm pore transwell (Millipore) were used to evaluate the cell invasion. Cells (1 × 10^5^) were dispensed into upper chamber with 100 μl fresh medium, while 600 μl normal serum-containing culture medium was placed in the under chamber. A cotton swab was used to remove the gel and cells in the upper chamber after 48 h of incubation and cells that invaded to the lower membrane of the chamber were fixed using 4% paraformaldehyde, stained with 0.1% crystal violet dye (Beyotime Institute of Biotechnology, Shanghai, China), and counted (at ×200 magnification) in five microscopic fields.

### In vivo experiments

Approximately 5 × 10^6^ HCCLM3, HCCLM3-miR-501-3p, HCCLM3-miR-501-3p-LIN7A, PLC/PRF, PLC/PRF/5-anti-miR-501-3p, or PLC/PRF/5-anti-miR-501-3p-shLIN7A cells were suspended in 100 μl of serum-free Dulbecco’s modified Eagle’s medium and then subcutaneously injected into the upper left flank region of the BALB/c nude mice. Four weeks after injection, the subcutaneous tumors were cut equally into small pieces (1 × 1 × 1 mm^3^/piece) and transplanted respectively into the liver of nude mice. Approximately 6 weeks later, transplanted mice were sacrificed and the volume of tumors was measured by using the following formula: (length × width^2^)/2^35^. The paraffin-fixed lung tissues were serial sectioned and stained with hematoxylin–eosin staining to determine lung metastasis.

### Luciferase assay

Cells were suspended in 24-well plates and cultured for 24 h. In all, 100 ng of wt or mt 3′-UTR of LIN7A vector was transfected into HCC cells using the Lipofectamine 2000 reagent (Invitrogen, USA) according to the manufacturer’s protocol. Cells were harvested and luciferase reporter gene assay was performed by the Dual Luciferase Reporter Assay Kit (Promega) in accordance with the manufacturer’s protocol after 48 h incubation.

### mRNA-seq

Trizol reagent (Invitrogen, CA, USA) was used to extract total RNA from cells. Magnetic oligo (dT) beads were used to purify RNA samples. mRNA samples were further reverse transcribed into first-strand cDNA, followed by the synthesis of a second cDNA. Fragmented DNA samples were blunt ended and adenylated at the 3′ ends. Adaptors were ligated to construct a library. DNA was quantified by Qubit (Invitrogen, CA, USA). Sequencing was carried out by an Illumina HiSeq 3000 SBS instrument. The expression of transcripts was calculated by fragments per kilobase of transcript per million fragments mapped. DESeq software was used to calculate the gene transcripts between different samples.

### Statistical analysis

SPSS software 16.0 was used for statistical analyses. Data were expressed as the mean ± standard deviation. Student’s *t*-test was used to compare quantitative data between experimental groups. Chi-square test was used to analyze categorical data. Kaplan–Meier survival analysis was used to compare OS and cumulative recurrence of HCC patients on the basis of miR-501-3p expression with statistical *P-*values evaluated by log-rank test. Pearson’s correlation analysis was performed between miR-501-3p expression and the mRNA levels of LIN7A. *P* < 0.05 was considered as significant.

## References

[1] Torre LA (2015). Global cancer statistics, 2012. CA Cancer J. Clin..

[2] Forner A, Llovet JM, Bruix J (2012). Hepatocellular carcinoma. Lancet.

[3] Bartel DP (2004). MicroRNAs: genomics, biogenesis, mechanism, and function. Cell.

[4] Pillai RS (2005). Inhibition of translational initiation by Let-7 MicroRNA in human cells. Science.

[5] Zhou SL (2016). miR-28-5p-IL-34-macrophage feedback loop modulates hepatocellular carcinoma metastasis. Hepatology.

[6] Kumar A (2011). MicroRNA in HCV infection and liver cancer. Biochim. Biophys. Acta.

[7] Ji J (2009). MicroRNA expression, survival, and response to interferon in liver cancer. N. Engl. J. Med..

[8] Budhu A (2008). Identification of metastasis-related microRNAs in hepatocellular carcinoma. Hepatology.

[9] Coulouarn C, Factor VM, Andersen JB, Durkin ME, Thorgeirsson SS (2009). Loss of miR-122 expression in liver cancer correlates with suppression of the hepatic phenotype and gain of metastatic properties. Oncogene.

[10] Wong QW (2008). MicroRNA-223 is commonly repressed in hepatocellular carcinoma and potentiates expression of Stathmin1. Gastroenterology.

[11] Furuta M (2010). miR-124 and miR-203 are epigenetically silenced tumor-suppressive microRNAs in hepatocellular carcinoma. Carcinogenesis.

[12] Su H (2009). MicroRNA-101, down-regulated in hepatocellular carcinoma, promotes apoptosis and suppresses tumorigenicity. Cancer Res..

[13] Ma S (2010). miR-130b promotes CD133(+) liver tumor-initiating cell growth and self-renewal via tumor protein 53-induced nuclear protein 1. Cell Stem Cell.

[14] Pineau P (2010). miR-221 overexpression contributes to liver tumorigenesis. Proc. Natl. Acad. Sci. USA.

[15] Meng F (2007). MicroRNA-21 regulates expression of the PTEN tumor suppressor gene in human hepatocellular cancer. Gastroenterology.

[16] Wong QW (2010). MiR-222 overexpression confers cell migratory advantages in hepatocellular carcinoma through enhancing AKT signaling. Clin. Cancer Res..

[17] Li Y (2004). Stepwise metastatic human hepatocellular carcinoma cell model system with multiple metastatic potentials established through consecutive in vivo selection and studies on metastatic characteristics. J. Cancer Res. Clin. Oncol..

[18] Murakami Y (2006). Comprehensive analysis of microRNA expression patterns in hepatocellular carcinoma and non-tumorous tissues. Oncogene.

[19] Wang R (2013). MicroRNA-195 suppresses angiogenesis and metastasis of hepatocellular carcinoma by inhibiting the expression of VEGF, VAV2, and CDC42. Hepatology.

[20] Zhang L (2013). MicroRNA-657 promotes tumorigenesis in hepatocellular carcinoma by targeting transducin-like enhancer protein 1 through nuclear factor kappa B pathways. Hepatology.

[21] Chuang KH (2015). MicroRNA-494 is a master epigenetic regulator of multiple invasion-suppressor microRNAs by targeting ten eleven translocation 1 in invasive human hepatocellular carcinoma tumors. Hepatology.

[22] Zhou JN (2015). MicroRNA-125b attenuates epithelial-mesenchymal transitions and targets stem-like liver cancer cells through small mothers against decapentaplegic 2 and 4. Hepatology.

[23] Xiao S (2016). Actin-like 6A predicts poor prognosis of hepatocellular carcinoma and promotes metastasis and epithelial-mesenchymal transition. Hepatology.

[24] Jou J, Diehl AM (2010). Epithelial-mesenchymal transitions and hepatocarcinogenesis. J. Clin. Invest..

[25] Gregory PA, Bracken CP, Bert AG, Goodall GJ (2008). MicroRNAs as regulators of epithelial-mesenchymal transition. Cell Cycle.

[26] Lee M, Vasioukhin V (2008). Cell polarity and cancer--cell and tissue polarity as a non-canonical tumor suppressor. J. Cell Sci..

[27] Royer C, Lu X (2011). Epithelial cell polarity: a major gatekeeper against cancer?. Cell Death Differ..

[28] Martin-Belmonte F, Perez-Moreno M (2011). Epithelial cell polarity, stem cells and cancer. Nat. Rev. Cancer.

[29] Gruel N (2016). LIN7A is a major determinant of cell-polarity defects in breast carcinomas. Breast Cancer Res..

[30] Wittekind C (2006). [Pitfalls in the classification of liver tumors]. Pathologe.

[31] Zhou SL (2015). CXCR2/CXCL5 axis contributes to epithelial-mesenchymal transition of HCC cells through activating PI3K/Akt/GSK-3beta/Snail signaling. Cancer Lett..

[32] Llovet JM (2008). Design and endpoints of clinical trials in hepatocellular carcinoma. J. Natl. Cancer Inst..

[33] Zhou SL (2012). Overexpression of CXCL5 mediates neutrophil infiltration and indicates poor prognosis for hepatocellular carcinoma. Hepatology.

[34] Zhou S (2011). Tacrolimus enhances the invasion potential of hepatocellular carcinoma cells and promotes lymphatic metastasis in a rat model of hepatocellular carcinoma: involvement of vascular endothelial growth factor-C. Transplant. Proc..

[35] Wang L (2000). High-dose and long-term therapy with interferon-alfa inhibits tumor growth and recurrence in nude mice bearing human hepatocellular carcinoma xenografts with high metastatic potential. Hepatology.

